# An Aptamer-Functionalised Schottky-Field Effect Transistor for the Detection of Proteins

**DOI:** 10.3390/bios12050347

**Published:** 2022-05-18

**Authors:** Thomas Farrow, Siriny Laumier, Ian Sandall, Harm van Zalinge

**Affiliations:** Department of Electrical Engineering and Electronics, University of Liverpool, Liverpool L69 3GJ, UK; thomas.farrow@liverpool.ac.uk (T.F.); siriny.laumier@liverpool.ac.uk (S.L.)

**Keywords:** biosensors, thin film sensors, field effect transistors, aptamers

## Abstract

The outbreak of the coronavirus disease 2019 (COVID-19) in December 2019 has highlighted the need for a flexible sensing system that can quickly and accurately determine the presence of biomarkers associated with the disease. This sensing system also needs to be easily adaptable to incorporate both novel diseases as well as changes in the existing ones. Here we report the feasibility of using a simple, low-cost silicon field-effect transistor functionalised with aptamers and designed to attach to the spike protein of SARS-CoV2. It is shown that a linear response can be obtained in a concentration range of 100 fM to 10 pM. Furthermore, by using a larger range of source-drain potentials compared with other FET based sensors, it is possible to look at a wider range of device parameters to optimise the response.

## 1. Introduction

In December 2019, a number of unusual cases of pneumonia of unknown origin were reported in Wuhan in the Hubei province of China [1]. This observation was subsequently confirmed as the novel coronavirus (2019-nCov) and renamed severe acute respiratory syndrome coronavirus 2 (SARS-CoV2) by the International Committee on Taxonomy of Viruses [2]. In the two years since, nearly all countries have imposed lockdowns and restrictions upon their citizens to slow the spread of COVID-19 to manageable levels and prevent health services from being overwhelmed. While the severity of these lockdowns and restrictions varies between countries, governments that have deployed mass rapid testing of citizens have generally required less severe restrictions and have appeared to be able to manage outbreaks with fewer cases and fatalities. 

Traditionally the presence of viruses has been determined by culture followed by observation. The advantages of this technique are low-cost and high specificity, but problems arise in that the sample needs to be taken from the infected area. In order to address these issues, two different types of detection methods have been developed. The first is the so-called antigen testing. The second method is molecular based. The current gold standard for the detection of viruses is polymerase chain reaction (PCR) [3], which is used to amplify the quantity of species-specific genes unique to a disease. It is, however, time-consuming and requires dedicated laboratories, bespoke equipment and trained personnel, making it non-ideal for the level of mass testing that has been required during the COVID-19 outbreak. As such, there is a need to develop faster and simpler testing, whilst maintaining the selectivity and sensitivity offered by PCR.

Over recent years, the so-called immuno-field effect transistor (FET) has been demonstrated as a potential low-cost biosensor platform. An immuno-FET is a field-effect transistor in which the gate electrode is replaced by a layer of antibodies specific to the target protein [4,5]. Once the target is attached to the antibody, the charged areas of the protein cause an electrostatic change in the conduction channel of the FET and hence modulate the source-drain current. Such devices have been demonstrated for potential SARS-CoV2 detection [6,7,8,9,10,11,12,13,14,15]. 

Currently, a major limitation in the performance of immuno-FETs is the screening effect when employed in physiological fluids [16]. The ions present in the solution cause the formation of a double layer with a thickness equal to the Debye length. Any change in the charge distribution outside this layer will not affect the conduction channel of the FET. For a standard salt concentration of 0.1 M, the Debye length is approximately 1 nm. As the antibodies have a size of roughly 10 nm, the target protein cannot be detected by the immuno-FET. This has in part been overcome by the development of a new group of molecules. These so-called aptamers consist of short DNA, RNA or peptide strands [17,18]. Due to their conformation and charge distribution, aptamers are capable of very specific binding to individual proteins. They are significantly smaller than antibodies with the result that a protein bound via an aptamer to the conduction channel will create the required change in the electrical properties at a higher salt concentration. This in turn means that it becomes possible to use this system with physiologically relevant liquids. In addition, the use of aptamers is significantly cheaper and more stable than the use of antibodies.

The majority of aptamer-based sensors employ electrochemical methods of detection and have been in existence for over a decade [19,20,21]. These techniques have also been used for the detection of the spike protein of SARS-CoV2 [11,22,23,24]. However, more recently, aptamer facilitated binding has been used within the setting of a FET and has been proven to be effective for a wide range of molecular sizes. Such sensors can determine the presence of small molecules, but also large proteins and whole cells [25,26,27,28,29,30]. The materials of choice for these aptamer-FETs are low-dimensional semiconductors, such as nanowires, graphene and thin film oxides. While these provide a good platform with low limits of detection, they are inherently expensive to produce. However, to date, there are no reports of sensors for COVID-19 that utilise the combination of aptamers with a field effect transistor. Considering the volume of sensors needed to cope with an outbreak like COVID-19, minimisation of the production cost is highly relevant.

In this paper, we explore the feasibility of a sensor concept employing a simple undoped silicon, aluminium Schottky source/drain-contacted, field-effect transistor functionalised with an aptamer sequence; the fabrication of these devices is simple and cheap. Traditionally aptamer-functionalised transistors rely on measurement with a single source-drain potential to determine the target protein concentration dependency of the source-drain current. Our focus is to use a wide range of potentials to obtain a broader concentration dependency not only to optimise the response of the sensor, but also to learn more about the processes that are occurring in the binding of the protein by the aptamer. To prove the functionality of the sensor, the SARS-CoV2 spike protein has been used as the target. This is the first time that aptamers have been used in a field-effect transistor as a binding element for the spike protein of SARS-CoV2 in a low-cost FET concept. The other important aspect is the ability for lay people to perform the test. In the proposed sensor this is facilitated by the fact that after the initial absorption in liquid, the actual sensing occurs in air.

## 2. Materials and Methods

**Figure 1:**
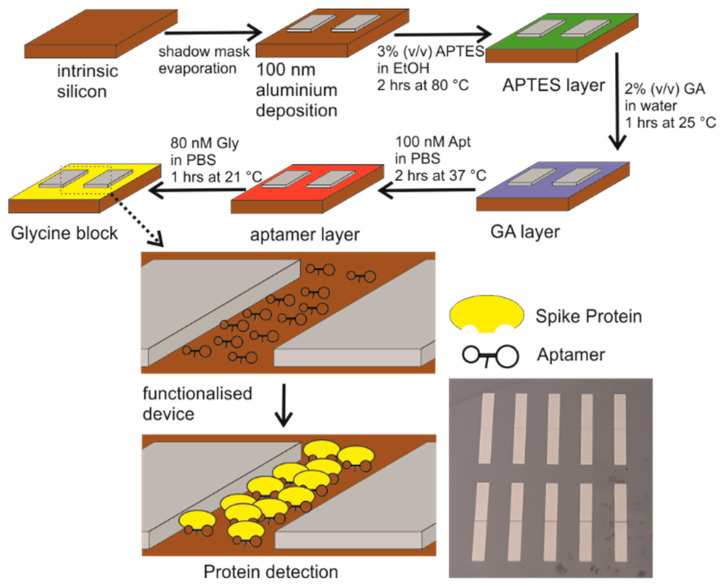
Schematic of the functionalisation process and subsequent detection of the spike protein. An aluminium contact is deposited on an intrinsic silicon sample. The device is functionalised with APTES, glutaraldehyde (GA), aptamer and glycine molecules in this order. The glycine is aimed at terminating any unbound aldehydes, which might create non-specific binding sites. In the final step, the process of detection is visualised inside the electrode gap. For clarity the aptamers and protein are only shown in the area between the electrodes; however, they are present everywhere on the silicon. At the bottom right is a photograph of the actual devices.

The devices were fabricated using nominally undoped silicon (University Wafer Inc., Boston, MA, USA). Aluminium Schottky source and drain contacts were employed to negate the need to implant junctions, which would significantly increase the cost of the device. Referring to the process flow diagram in Figure 1, 100 nm of aluminium (Puratronic 99.99% purity) was deposited by thermal evaporation using a Moorfields evaporator. The rectangular Schottky contact electrodes with dimensions of 1 × 3.5 mm were defined using a shadow evaporation mask. The drawn channel length is 100 µm and forms the sensor area of the device. 

The surface was functionalised in order to attach the binding element to the sensor area. All chemicals used in this process were obtained from Merck and used as-received unless otherwise stated. The aptamer probe molecule was attached to the silicon surface using a well-established silanisation method [13,14,31]. In brief, the wafer was immersed in an ethanol solution containing 3% (*v*/*v*) (3-aminopropyl)triethoxysilane (APTES) at 80 °C. After 2 h the sample was removed from the solution and washed four times with ethanol and deionised water to remove any excess material. This step was completed by drying the sample under N_2_ and a curing step of 1 h at 110 °C, which ensures the cross linking of the silanes. The surface at this point is terminated with an amine group that can be used for further functionalisation. 

The samples were then immersed in an aqueous solution containing 2% (*v*/*v*) glutaraldehyde (GA) at room temperature (25 ± 2 °C) for 1 h. The glutaraldehyde acts as a crosslinker, binding with the amine-terminated silane and providing an aldehyde binding group for the amine-terminated aptamer sequence. After rinsing with deionized water, the sample was dried under N_2_. The functionalised devices were subsequently immersed in a solution containing amine-terminated aptamers (Eurogentec, Seraing, Belgium). The device and solution (200 μL, 100 nM) were incubated at 37 °C for 2 h and then rinsed with 1× phosphate-buffered saline (PBS) and deionized water to remove the excess aptamers and subsequently dried under N_2_. An aptamer previously reported to bind to the spike protein of the SARS-CoV2 virus was used [32]. The specific sequence is 5′-CAGCACCGACCTTGTGCTTTGGGAGTGCTGGTCCAAGGGCGTTAATGGACA-3′ with an amine group attached to the 5′ end.

There is a possibility that not all aldehyde groups provided by the glutaraldehyde bound to an aptamer and as such, they would provide sites that potentially can bind non-specifically to any amine group present in the proteins. To prevent this, following the aptamer functionalisation, the samples were immersed in a PBS solution containing 80 mM glycine for 60 min at room temperature. They were subsequently rinsed in PBS and dried in an N_2_ atmosphere to remove excess material as well as any water in the layers.

Once the samples were functionalised, the response to varying concentrations of the recombinant SARS-CoV-2 spike protein, S1 Subunit [33] (Cambridge Bioscience, Cambridge, UK) was determined with serial dilutions of the spike protein in PBS. Prior to any characterisation, the samples were exposed to a PBS solution without the protein. For all concentrations of spike protein, the samples were left in the solution for 15 min. They were subsequently washed with PBS and blown dry with N_2_ in order to remove unbound protein and excess water. While the protein was deposited from PBS with a physiologically relevant ionic strength, the actual experiments were performed in air. In total fifteen devices over three separately fabricated samples were tested and the results presented here are considered to be representative of this set (see Supplementary Information Figure S2). 

Electrical characterisation was performed in air using a screened probe station with tungsten needles in combination with a Keysight 4155B Semiconductor parameter analyser.

## 3. Results

### 3.1. Basic Device Characterisation

In order to characterise the basic properties of the devices used, two measurements were performed. The first was an IV-characteristic between the front and back. The front contacts were made as previously described. The back contact was created by first sanding the back of the silicon wafer before depositing an aluminium contact to ensure that this is an Ohmic contact. The IV-characteristic, not shown, shows that the electrodes form a weakly rectifying contact.

**Figure 2:**
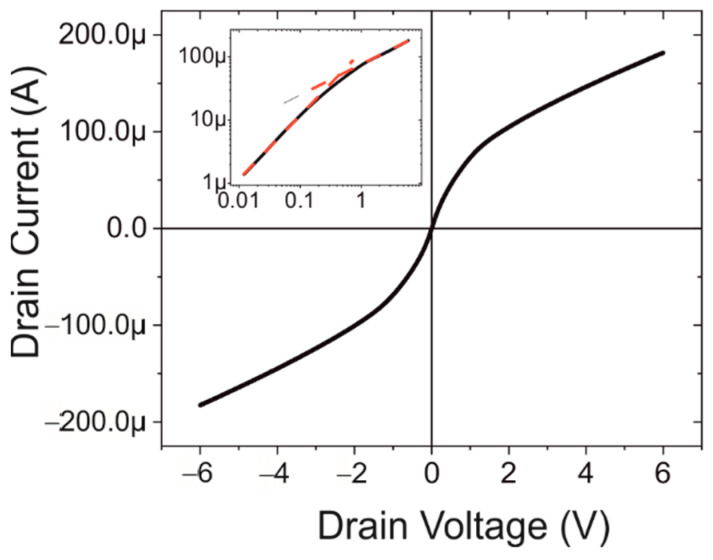
The IV-characteristics of the bare silicon device between the source and drain contact. The inset shows the log-log plot of the positive bias potentials. The red dotted lines indicate the slope, which is 1, indicating Ohmic behaviour at low potential and 0.5 at higher voltage.

**Figure 3:**
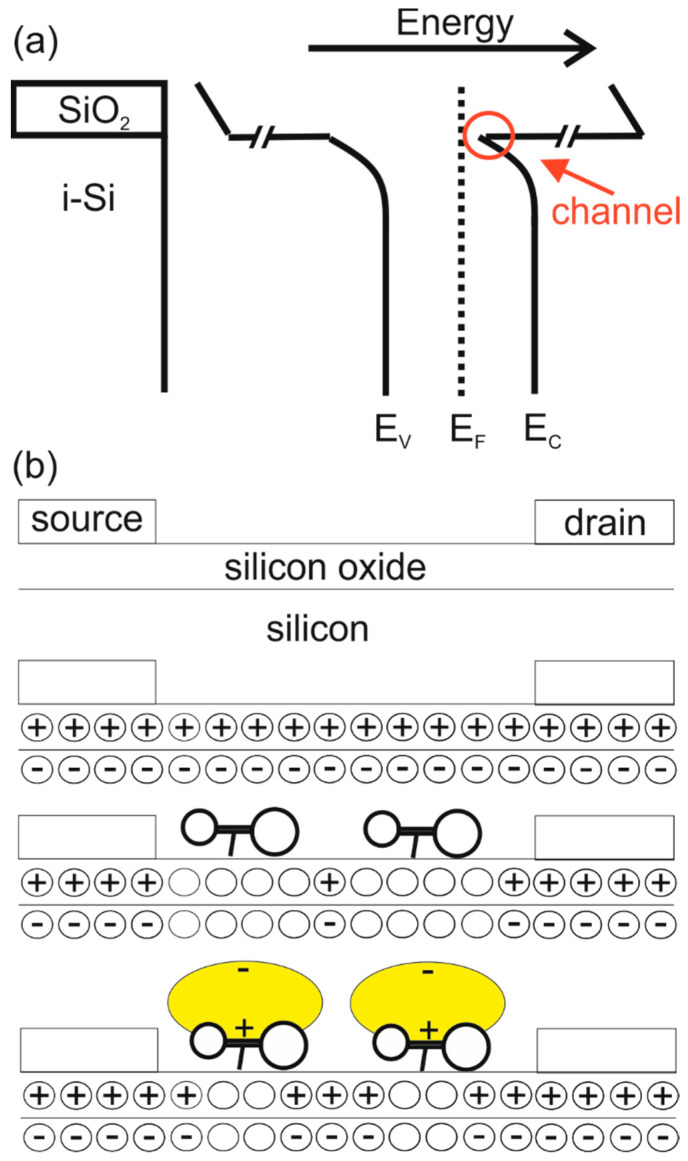
A schematic representation of the experiment. (**a**) shows the band diagram at the interface between the SiO_2_ and the Si; the formation of the channel due to the band-bending is indicated. In (**b**) the effect of the presence of the aptamers and protein is indicated. At the top, the general layout of the FET is shown. The steps below illustrate the major points in the experiment. The ‘empty’ circles represent equal amounts of electrons and holes, whereas an area with a pre-dominantly negative or positive charge is indicated with a minus or plus signs, respectively.

Figure 2 shows the IV-characteristics between the source and drain without the application of the functionalisation. It can be seen that the IV-characteristics are that of a field effect transistor. The creation of a channel is deemed to be due to the presence of the fixed positive charge in the native oxide (a well-known property of the Si/SiO_2_ system) and hence the channel is formed of electrons [34]. This is schematically depicted in Figure 3.

The inset in Figure 2 shows the data on a log-log plot. Two different regimes are apparent. The first regime at low voltages (<0.1 V) has a slope of approximately 1 indicating the expected Ohmic region of FET characteristics. This is followed by the final pinch-off region where the slope decreases to 0.5, the saturation region.

### 3.2. IV-Characteristics of Functionalised Devices

**Figure 4:**
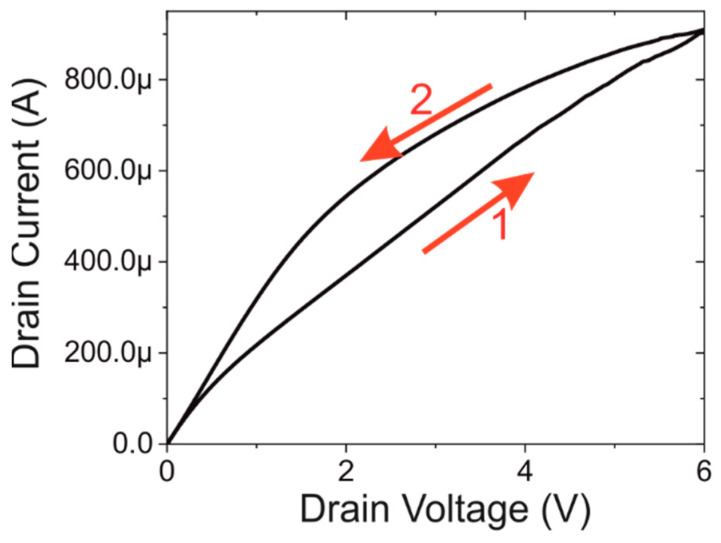
IV-characteristics of a functionalised device with a spike protein concentration of 1 pM. The arrows and numbers indicate the direction of the voltage sweep. During the upsweep (1) a FET type characteristic is observed, and the return sweep (2) show hysteresis caused by slow moving ions.

For the characterisation of the functionalised devices the focus is on the positive bias potentials. The IV-curves are fully symmetric as shown in the Supplementary Information, Figure S1, so for clarity, the negative part has been omitted. An example of the IV response of a functionalised device can be seen in Figure 4.

Upon the addition of the functionalisation layers, a distinct hysteresis can be observed in the IV-characteristics. Concentrating on the forward sweep labelled 1 in Figure 4, it can be seen that the basic characteristics of the curve are similar to those observed in Figure 2. This indicates that the conduction is still determined by the standard FET behaviour. In the ideal long-channel MOSFET model, the slope after saturation should be close to zero; however, the very low effective doping is thought to result in a degree of effective channel shorting.

The backward sweep labelled 2 in Figure 4 show distinctly different behaviour. The voltage sweep follows the arrows as indicated in the graph. It is proposed that the observed effect can be explained by slow-moving (low mobility) ions present on the surface [35]. During the initial sweeps starting from 0 to ±6 V, ions present in the organic film created in the functionalisation and subsequent steps move under the action of the electric field. Modulation of ions close to the source electrode can cause a corresponding change in the electric field, which enhances charge injection. It is apparent that such movement serves to enhance the injection of charge carriers, and hence the current, on the reverse sweep.

### 3.3. Protein Dependence

**Figure 5:**
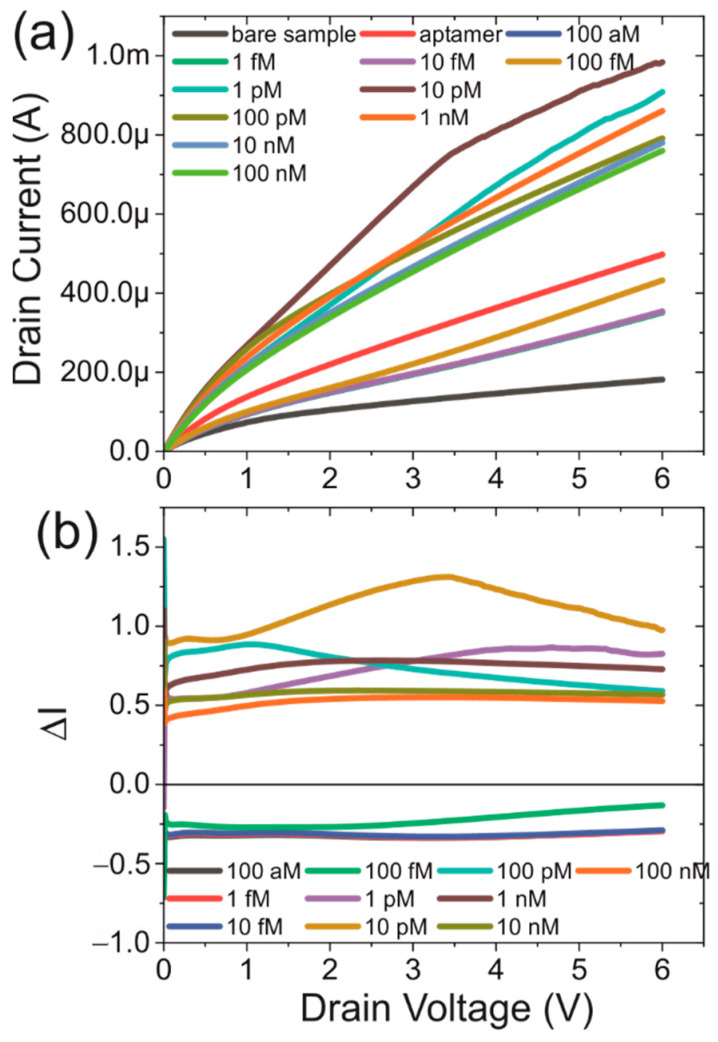
(**a**) IV-characteristics of a functionalised device with at varying spike protein concentrations (up sweep only). (**b**) The relative change of the current as a function of bias potential.

The IV-characteristics at various concentrations of the spike protein ranging from 0 to 10 nM in PBS are shown in Figure 5a. The return sweeps affected by the hysteresis are omitted for clarity. However similar behaviour as discussed above is seen at all concentrations. The curves here are representative for 15 devices spread over 3 independently fabricated samples. While these curves in themselves do show that there is a significant change in the electronic properties due to the presence of varying amounts of protein, a better parameter is required.

We introduce a parameter, Δ*I*, to facilitate analysis of the output without the need to account for minor fluctuations due to the fabrication process. The parameter is defined as the relative change in the current and is given in Equation (1) below:

(1) $$\Delta I=\frac{I-I_{0}}{I_{0}},$$

where *I*, is the measured current and *I*_0_ is that measured at the same voltage in the IV-characteristic without the presence of the target protein labelled ‘aptamer’ in Figure 5a. Shown in Figure 5b are the ratios over the complete source-drain voltage range. It can be seen that they are relatively uniform over the whole potential range. At several protein concentrations around 1 pM, the behaviour is slightly different and this will be discussed later.

The data shown in Figure 5b can provide information regarding the charges inside the aptamer and protein layers. Upon the addition of the aptamer, the current at a fixed potential is significantly increased compared with that of a bare sample. This reduction indicates the presence of a net positive charge in the aptamer closest to the channel. Upon the addition of the proteins, the current initially decreases before it displays a significant increase. This is most likely caused by the initial shielding of the charge in the protein before the positive charge in the protein becomes the major charge to affect the channel.

**Figure 6:**
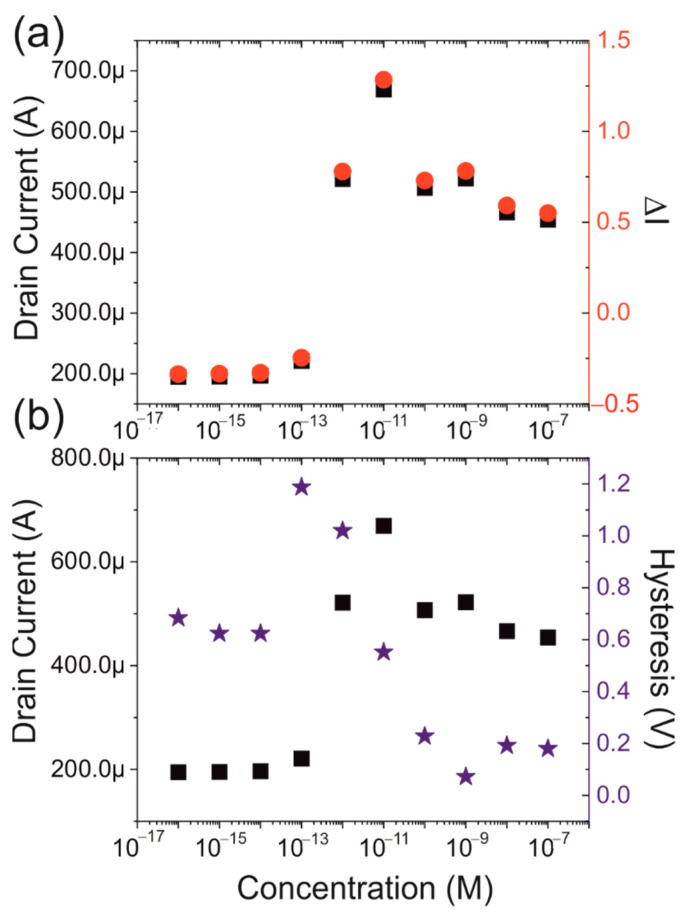
(**a**) comparison between the current at +3 V (black) and the relative change (red). (**b**) comparison between the current (black) and the hysteresis (blue).

The values for both the current and Δ*I* at ±3 V are shown in Figure 6a. A lateral shift can be seen between the positive and the negative voltage plots; however, the same linear behaviour is apparent. At low concentration, up to 100 fM, there is insufficient sensitivity to detect the presence of the spike protein. At concentrations above 10 nM, the sensor saturates. However, between these values there is a concentration range in which the device shows a linear response to changes in concentration.

Referring to Figure 6a, the current increases within the linear range as (224 ± 44) µA, per decade increase of the concentration with a Pearson correlation of 0.99. In all cases, the values are given as the mean ± one standard deviation. Similar values have been found for the other devices that were examined and the result can therefore be considered typical. The ratio changes at a rate of approximately 0.76 (±0.15) per decade change in the concentration, indicating that the aptamer functionalised field-effect transistors have the potential to act as a sensor capable of detecting spike protein.

A consistent observation that can be made is that there appears to be an overshoot. A basic interpretation would be that each protein added to the sensor will add additional charge to the channel and hence increase the current flow. This effect ceases when the protein film saturates and no further proteins can be added. However, this does not adequately explain the overshoot. The most likely cause for this is that the protein film undergoes some type of reorganisation which reduces the amount of charge close to the channel. This reduces the number of charge carriers in the silicon and hence reduces the current flow.

In Figure 4 it is shown that all IV-characteristics show a significant amount of hysteresis. To be able to characterise this, the hysteresis will be defined as the voltage difference between the up and down sweep at half the maximum current observed at +6 V. In Figure 6b, this parameter is plotted and compared with the current at +3 V. It can be seen that they are almost opposites of each other. We suggest that, at low protein concentrations, slow-moving ions from the buffer solution that contains the protein, are trapped in the layers formed by the aptamers. As they are weakly bound to the charge in the aptamer, they can remain there during the washing steps. However, when an electric potential is applied to the drain contact, the ions will start to slowly move in accordance with the electric field. This results in variations in the electric field on the back sweep, as shown by the hysteresis. Once the protein concentration reaches a certain threshold, 100 fM, the protein will assist this effect by trapping additional ions, which increases the observed hysteresis. When the protein concentration is increased further, the protein interaction with the aptamer will reduce the number of ions that can be trapped within the layer. This results in the observed reduction of the hysteresis. At around 1 nM protein concentration, saturation occurs and no further changes are observed.

**Figure 7:**
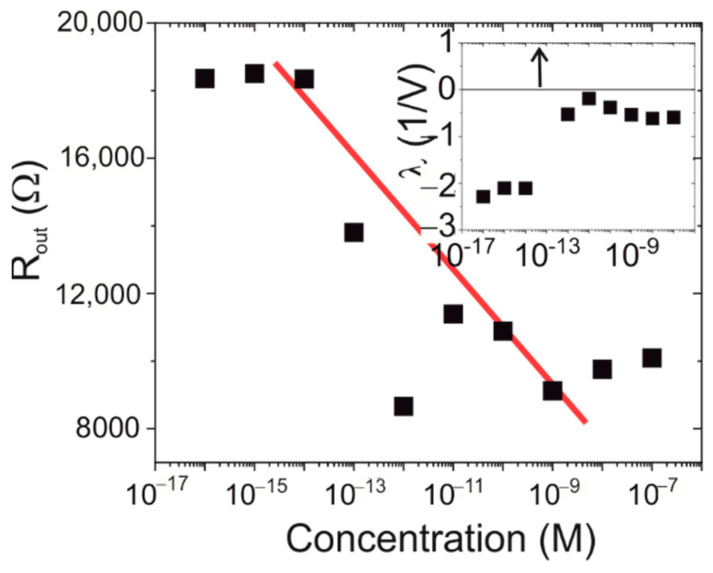
The output resistance as a function of the spike protein concentration. The red line indicates the trend line. The inset shows the lambda parameter which is related to the channel shortening. The arrow at 100 fM indicates an omitted point at λ = 33.4 V^−1^.

The final part of the IV-characteristics is the non-zero slope in the saturation region. This slope defines the output resistance of the transistor and the protein concentration dependence is shown in Figure 7. During the low concentrations where the amount of protein present on the surface is insufficient to create a change in the electrical properties of the transistor, the output resistance is constant. It then shows a linear decrease of (1.6 ± 0.3) kΩ per decade concentration until the saturation occurs at 1 nM and the output resistance levels remain constant. The output resistance is partly caused by the channel shortening of the transistor itself, which will significantly change the basic device properties. 

The channel shortening is characterised by the parameter λ, which is the slope of the characteristic and is shown in the inset of Figure 7. As can be seen, at low concentration it is constant, and this is followed by a significant increase before returning to a lower value which is about half the value at low concentrations. This pattern suggests that the device goes through some significant changes associated with the presence of the protein as indicated by the changes in the channel shortening.

### 3.4. Controls

**Figure 8:**
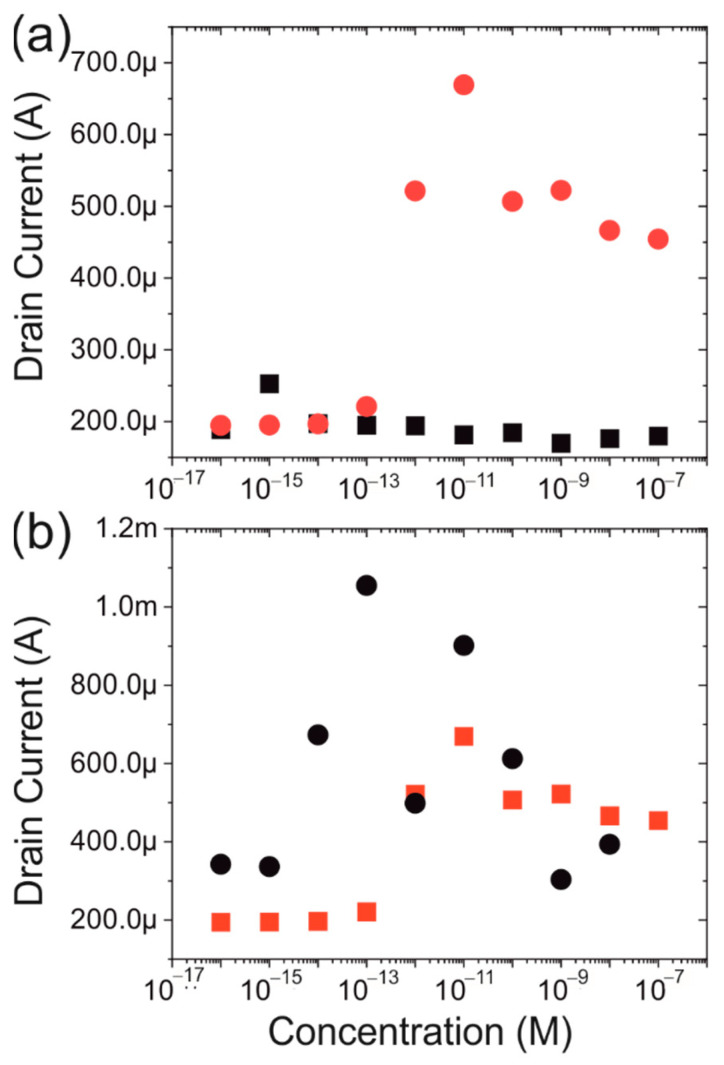
(**a**) comparison between the current at +3 V for BSA (black) and spike protein (red). (**b**) comparison between the current for spike protein in artificial saliva (black) and PBS (red).

Final tests were conducted to ensure that the selectivity is accurate and that the sensor only detects the presence of the protein under study. In Figure 8a, the ratio is compared between the samples that are prepared with spike protein and with bovine serum albumin (BSA). It can be seen that the devices show no response to the presence of an increasing concentration of BSA.

A further control experiment was conducted as follows. A concentration dependence experiment was performed with the spike protein dissolved in artificial saliva compared with a PBS buffer solution. The artificial saliva mimics the proteins and molecules typically found in saliva and thus presents a close approximation of a mouth swab. As can be seen in Figure 8b, the results are noisier than those obtained from using a PBS solution, however, the generally observed trend can still be found.

## 4. Discussion

In Figure 5a,b, a number of IV-spectra for different concentrations of spike protein intersect each other. Under the assumption that the effect of the protein is purely one that changes the charge density in the channel of the FET, this should not be possible. However, in control experiments using the same procedure but using a glass substrate, a significant current was observed after functionalisation, increasing further at high protein concentrations (see Supplementary Information, Figure S3). The result is that the current system is a combination of two FETs in parallel that provide a gate to each other. These effects have been reported previously [36] and have been modelled for few layer 2D materials [37] such as graphene. Although this predicts a change in the behavior of the FETs compared with an individual FET, there are a number of differences with the system studied here. The first is that the reported structures are identical, but here there are two completely different conductive layers, Si and the functionalisation/protein layer. The second difference is that the conductivity of the functionalisation/protein layer is dependent on the protein concentration and distribution and cannot be considered to be uniform. As a result, it can be expected that the presence of the second FET will affect the observed IV-characteristics, but the exact effect of this is unknown. The observed intersecting patterns in Figure 5 coincide with those concentrations where anomalous effects are observed in the various other parameters that were examined.

When comparing the experiment performed here with those that have been reported previously, several observations can be made. A number of papers have been published in the area of FET-based sensors using aptamers as the recognition element [25,28,29,30]. All of them report lower limits of detection compared with this study. The reported response time is of the same order of magnitude. However, the studies published to date, in contrast to the work here, are focused on using 1- and 2-dimensional material, for example nanowires and graphene. In this paper, the emphasis is on obtaining the sensing element while using inherently cheaper materials; here we used basic silicon without any further processing. A further difference is that, in contrast to the in-air analysis that is performed here, the reports to date are all performed in liquid. While in-liquid analysis has advantages due to the measure of control over the gate potential, it adds an additional complexity to the use of the sensory system. A second comparison can be made with recent papers that use a FET as the sensor to detect spike protein. As above, the majority of the papers report using low-dimensional materials [25,27,28,29,30]. However, a second more substantial difference is that all the spike protein FET sensors reported to date use antibodies to create the selectivity. As a consequence, the effect of the charge of the protein on the FET is reduced. This has resulted in comparable responses to the presence of spike protein. Although further steps need to be performed to optimise the time required to obtain a result from the test, it currently is comparable to both lateral flow tests [38] as well as those reported for other aptamer based FET-sensors [9]. 

In comparison to other reported experiments, the proposed device has the great advantage of very low-cost manufacturing, which is the main driver for mass commercial, point-of-care usage. The proposed sensor utilises the natural oxide on an as-received wafer without the need of any further fabrication steps to create the field effect transistor, apart from the deposition of aluminium contacts. This makes the fabrication process inherently cheaper. An additional cost advantage is the in-air analysis of the sensor. This facilitates its use in a point-of-care setting as it does not require the use of laboratory space, equipment and/or personnel, which leads to significant cost savings in the processing of samples.

It is recognised that in order to translate this work into a commercial product, additional steps would need to be undertaken. In the current work, synthetic spike protein has been studied and this protein is part of the membrane of the actual virus. The first step, therefore, is to translate the concentration range as observed here to the concentration of viruses. The next step is the verification of the selectivity. The selectivity experiments need to be performed to ensure that the device is solely responsive to the target, in this case, the spike protein. While this has been addressed here by selectivity experiments with BSA as well as artificial saliva, further verification in actual saliva or nasal swabs would be required. A further complication may arise when mutations in the SARS-CoV2 virus causes significant changes within the spike protein to such an extent that the current aptamer sequence no longer binds to the novel spike protein. However, this difficulty will be similar for all sensors. In addition, the sensitivity of the proposed sensor is crucial. Ideally, the presence of a single SARS-CoV2 spike protein should trigger a positive response. However, it is difficult to estimate the charge associated with a single protein so the ultimate sensitivity cannot be assessed at this stage. It is conceivable that a nanowire-based technology could produce the ultimate sensitivity. Note that our aim here is merely to show the feasibility of an aptamer-FET to detect spike protein and these optimisation steps are deferred to future research. Furthermore, as the spike protein is considered in isolation within this paper, a direct comparison of the sensitivity with real-life samples where the spike protein is part of the virus membrane is not possible.

In previous studies of aptamer-based FETs, the sensing was performed at a single source-drain potential and by varying the applied additional gate potential [8,16]. Here a complete IV-characteristic was studied which allows for further characterisation of the properties of the transistor. In the aptamer-FETs that have been reported to date [25,27,28,29], the source drain potentials have been restricted to remain well below the saturation region, excluding parameters associated with the saturation, such as the channel shortening and output resistance, from playing a part in the analysis of the protein concentration. Considering the results presented here, the output resistance of the transistor shows the best response to variations in the protein concentration with respect to the curve uniformity and lack of observed overshoots. 

## 5. Conclusions

In summary, using a known aptamer sequence that binds to a spike protein, we have shown that it is feasible to successfully detect the presence of a specific protein. The silicon field-effect transistor sensor exhibits a linear detection range between 100 fM and 10 pM or 75 pg/mL to 7.5 ng/mL. The sensor is fabricated by a very low-cost, simple process, and the ultimate sensing occurs in air. The study further demonstrates the feasibility of using various transistor properties as parameters for the sensing element with the output resistance being the most promising.

## Data Availability

The data used within this study is available from the authors.

## Supplementary Materials

The following supporting information can be downloaded at: https://www.mdpi.com/article/10.3390/bios12050347/s1, Figure S1: Full IV-curve; Figure S2: All sample analysis; Figure S3: concentration dependence on glass substrate.
