# Supplementary material for: An Aptamer-Functionalised Schottky-Field Effect Transistor for the Detection of Proteins
Source: Biosensors (Basel). 2022 May 18;12(5):347. doi: 10.3390/bios12050347 (PMC9138399; doi:10.3390/bios12050347)
Supplement: Supplementary file 1 [file biosensors-12-00347-s001.zip › biosensors-1669795-supplementary.pdf]

An aptamer functionalised Schottky-field effect transistor for the detection of proteins

Thomas Farrow, Siriny Laumier, Ian Sandall and Harm van Zalinge

Supplementary Information

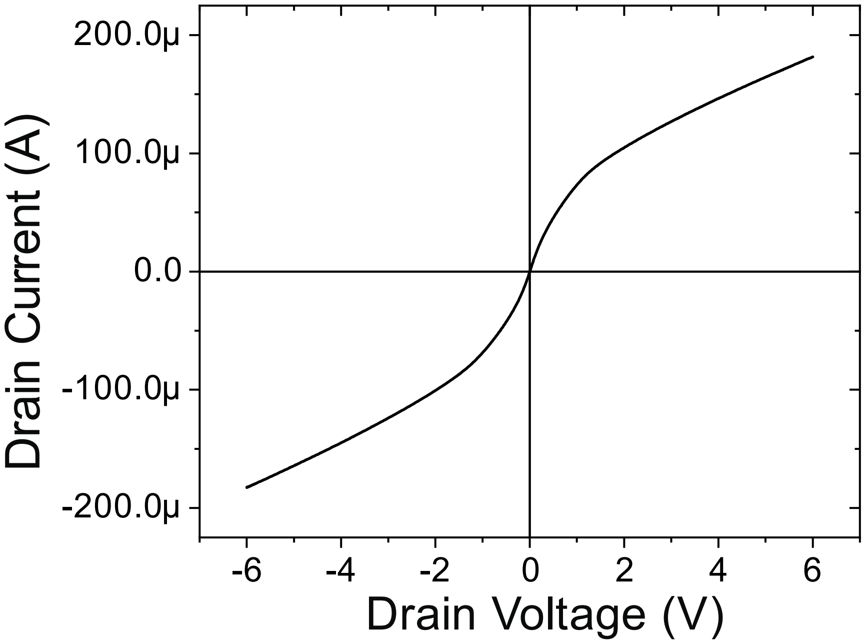

Figure S1. An example of the full IV-curve, which shows that the positive and negative source-drain potentials are symmetric.

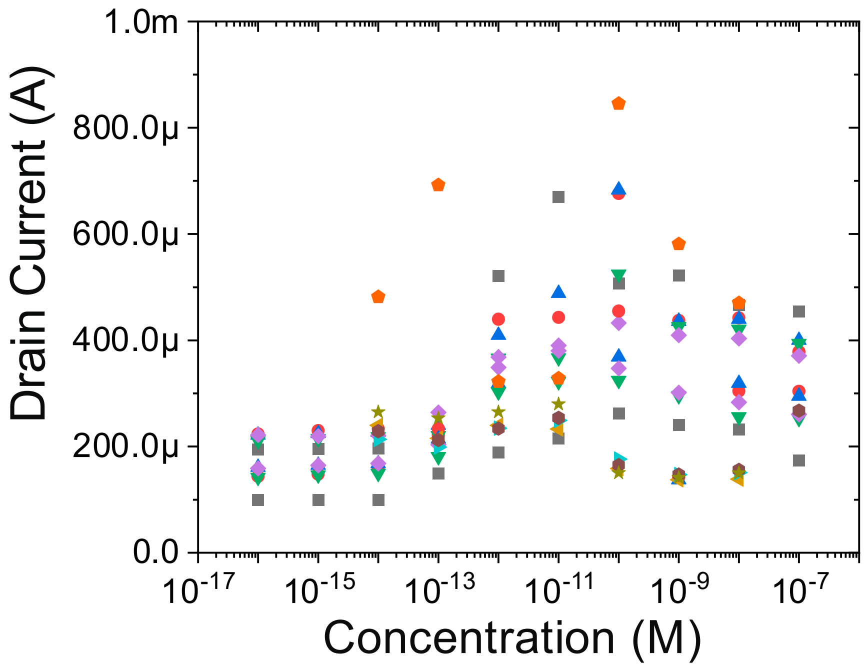

Figure S2. The concentration dependency of the various devices used in this study. The measure used is the current at +3 V of the source-drain potential. The trend of the increase in current between 100 fM and 10 pM are consistent across the samples. Each colour/ symbol represents a different device.

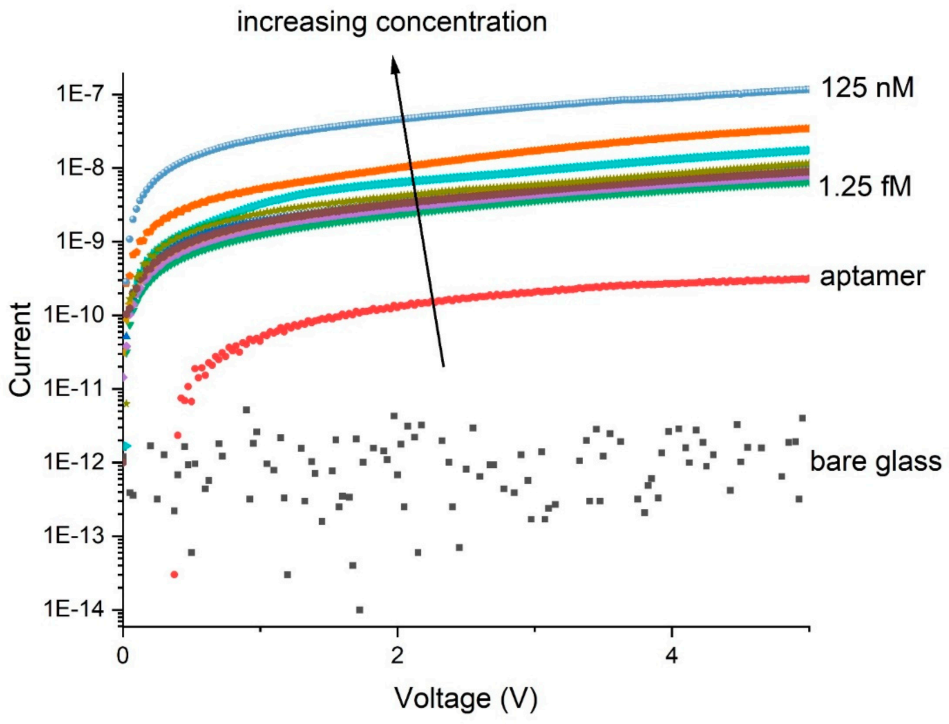

Figure S3. The concentration dependence of a device made on glass instead of silicon, which shows that there is a significant current flowing between the source and drain through the organic layer.
